# To Identify the Important Soil Properties Affecting Dinoseb Adsorption with Statistical Analysis

**DOI:** 10.1155/2013/362854

**Published:** 2013-04-22

**Authors:** Yiqing Guan, Jianhui Wei, Danrong Zhang, Mingjuan Zu, Liru Zhang

**Affiliations:** ^1^State Key Laboratory of Hydrology-Water Resources and Hydraulic Engineering, Hohai University, Nanjing 210098, China; ^2^Institute of Meteorology and Climate Research, Karlsruhe Institute of Technology (KIT), 82467 Garmisch-Partenkirchen, Germany; ^3^National Center for Computational Hydroscience and Engineering, University of Mississippi, Oxford, MS 38655, USA; ^4^Nanjing Hydraulic Research Institute, Nanjing 210029, China

## Abstract

Investigating the influences of soil characteristic factors on dinoseb adsorption parameter with different statistical methods would be valuable to explicitly figure out the extent of these influences. The correlation coefficients and the direct, indirect effects of soil characteristic factors on dinoseb adsorption parameter were analyzed through bivariate correlation analysis, and path analysis. With stepwise regression analysis the factors which had little influence on the adsorption parameter were excluded. Results indicate that pH and CEC had moderate relationship and lower direct effect on dinoseb adsorption parameter due to the multicollinearity with other soil factors, and organic carbon and clay contents were found to be the most significant soil factors which affect the dinoseb adsorption process. A regression is thereby set up to explore the relationship between the dinoseb adsorption parameter and the two soil factors: the soil organic carbon and clay contents. A 92% of the variation of dinoseb sorption coefficient could be attributed to the variation of the soil organic carbon and clay contents.

## 1. Introduction

Dinoseb (2-*sec*-butyl-4,6-dinitrophenol) is a member of the dinitrophenol family of pesticides, commonly used for controlling the growth of annual grassy and broadleaf weeds. It has long persistence, which leads to an accumulation in soil. It has been found in many areas of the world [1–3]. Many countries have prohibited the usage of the dinoseb. In USA, EPA banned dinoseb usage in 1986. Much research focuses on dinoseb's toxic effects on human beings, animals, and microorganisms [4–6]. And the measuring technique has also well been studied [7–10].

After being applied to soil, the transport and fate of herbicides are controlled by many complicated mechanisms, including sorption to soil, uptake by plants, transport vial runoff and leaching, biodegradation, photodegradation, volatilization, and chemical degradation [11, 12]. Sorption is one of the most important mechanisms that influence the presence of herbicides in soil [12]. To evaluate the sorption property of herbicides, the popular methods include batch equilibrium technique, column experiments, and field experiments. Comparing with other two techniques, batch experiments are easy and fast to perform, and the cost is low [13]. The results of the batch sorption equilibrium experiments are usually fitted with linear sorption model or Freundlich sorption model to derive the sorption parameters. 

Since the sorption of herbicides to a large extent was determined by soil properties, such as organic carbon (OC) content [14, 15], clay content [16, 17], and pH value [18], the multiple regression is usually applied to explore the relationships between the sorption parameters and soil environmental factors. However, in multilinear regression, if the predictor variables are not independent, multicollinearity will be a common statistical phenomenon. Multicollinearity may not affect the goodness of the multiple regression prediction, but it reflects the determination of the importance of each environmental factor. Stepwise regression can be used to correct for multicollinearity. It has been frequently applied in educational and psychological research, both to select useful subsets of variables and to evaluate the order of importance of variables [19]. Path analysis was developed around 1918 by Sewall Wright. It was usually used to decompose correlations into different pieces for interpretation of effects. The two methods have been applied to many studies, including biology, sociology, and econometrics [20, 21]. The objectives of this study, therefore, are (1) to use the bivariable correlation analysis and path analysis to investigate the extent of these influences and explicitly explain direct and indirect effects of soil characteristic factors on dinoseb adsorption, (2) to use stepwise regression analysis for excluding the factors which have little influence on the adsorption parameter, and (3), based on these results, to set up regression equation of adsorption values with the most important soil factors. 

## 2. Materials and Methods

### 2.1. Soil Sample Collection

The soil samples were collected in the upper Rhone river valley in Southwest Switzerland. Dinoseb had been found in the groundwater of the Rhone plain. This alluvial plain is well cultivated and of great economic and ecological importance, but it was alleged a high vulnerability of the groundwater to contamination [22]. Along a transect, pits A, B, C, and D were excavated; the distances between the pits were 6.5 m, 8.1 m, and 6.5 m, respectively. At these four sites, altogether 55 small disturbed soil samples were collected at 15, 30, 55, 70, and 85 cm depths.

### 2.2. Characteristics of Dinoseb

The purity of the dinoseb production used in the study was 93% (Dr. Ehrenstorfer, Germany). The properties of dinoseb are summarized in Table 1.

### 2.3. Experimental Design

First of all, basic soil properties such as bulk density, porosity, particle size distribution, pH, cation exchange capacity (CEC), and organic carbon content were determined.

Soil samples collected from the field were air-dried at room temperature and sieved at 2 mm. Then 3 g of dry soil was mixed with 6 mL of dinoseb solutions at different concentrations into a 9 mL polypropylene centrifuge tube. The concentrations are 0, 1.5, 4.5, 9, and 15 mg/L, respectively. The tubes were shaken for 24 hrs on a rotary tumbler at 20°C. This duration was sufficient to achieve sorption equilibrium, but not long enough for chemical or biological transformations to significantly affect the results, as attested by sorption kinetics tests. The aqueous phase was separated from the solid phase by centrifugation for 15 min at 7000 rpm. The supernatant was filtered through a disposable 0.45 *μ*m cellulous filter. The filtrates were analyzed by injection into a high performance liquid chromatography with diode array detection (Hewlett Packard series 1050) using a C18 column of 25 cm length (VYDAC). The light absorption wavelength used for the detection of dinoseb is 265 nm. The flow was set as 1 mL/min; the solvents used for HPLC were distilled water, acetonitrile (purity ≥ 99.8%), and 0.05% trifluoroacetic acid. The original composition of flow was 40% acetonitril, 40% water, and 20% trifluoroacetic acid. During each measuring, trifluoroacetic acid was kept as constant as 20%; acetonitrile increased from 40% to 65% over the first 15 min, then increased to 80% over the next 5 min after this measuring started; acetonitrile then decreased back to 40% over 5 min and sustained at this level until the end of this measuring, which was 30 min after measuring starts. 

The adsorbed dinoseb mass was calculated with the difference between the initial dinoseb concentration and that measured in the supernatant. All batch sorption experiments were conducted in triplicate.

### 2.4. Statistical Analysis

Correlation analysis and path analysis in this study were used to demonstrate the degrees of the variables' interactions or interferences with each other and the exact variable with the most exerting influence. Stepwise multiple-linear regression analysis was used for identifying the linear relationship between dinoseb absorption coefficients with soil properties. Significance of differences was either tested by using a parametric *t*-test or *F*-statistics in ANOVA (analysis of variance). 

Stepwise multiple-linear regression [24] is one method in multiple linear regressions that used to analyze the linear relationship between single dependent variable with several independent variables. It was selected for this research because (1) multiple-linear regression makes use of the most of the directly observed and experimental information that has been available [25]; (2) the number of controlled variables (OC, CEC, pH, Clay) is fairly small so that it could be easily performed to analyze including all of them; (3) the bivariate correlations among soil properties with the dinoseb adsorption values are not explicitly fixed especially with the influence of multicollinearity; (4) the problem of overfitting could be avoided by adding or deleting variable with the specific criteria. Therefore, backward elimination [26] is applied to build up the final regression equation describing a predicted variable as a function of several independent variables. It follows these procedures: firstly adding all the independent variables into regression, secondly analyzing significance of difference about the partial coefficient of each independent variable and deleting the one with lowest significant contribution to the regression equation compared with the removing criteria (alpha-to-remove value), and finally repeating the regression modeling and testing with remaining variables and removing until all the remaining variables have significant contribution to the regression equation. But some issues of stepwise regression still exist such as that it cannot explicitly interpret the multicollinearity between controlled variables [27].

Due to the problem of multicollinearity in regression [28, 29], before setting up a stepwise multilinear regression, bivariate correlation analysis and path analysis [30] based on the causal relationship were adopted to make explicit the rational of conventional regression calculations. Path analysis have special usefulness in decomposing the soil property effects on the dinoseb adsorption into direct and indirect effects and quantifying the collinearity in the regression model. Note that the direct and indirect effects importantly depend on how the model is built [31]. In this study, only the regression model which includes all variables was applied to path analysis to capture the overall direct and indirect effect from four soil properties on the dinoseb adsorption values.

## 3. Results and Discussion

### 3.1. Sorption Isotherms

The physical and chemical properties of the soil samples collected at four sits over 5 depth were summarized in Table 2. 

Fifty-five sorption isotherms of dinoseb are determined. The isotherms have been fitted by Freundlich model:

(1) $$\begin{array}{c} S=K_{f}C^{n}, \end{array}$$

where *S* is the adsorbed chemical concentration (g/g), *K*_*f*_ is the Freundlich partition coefficient, (cm^3^/g), *n* is an empirical coefficient and *C* is the equilibrium concentration (mg/L).

Thirty out of the 55 dinoseb sorption isotherm fittings have a *R* ^2^, more than 0.95. The *R* ^2^ values of 19 fittings are from 0.90 to 0.95. The left 6 fittings have a *R* ^2^ value from 0.87 to 0.90. Therefore, the model can well describe most of the dinoseb sorption at concentrations less than 15 mg/L. The derived Freundlich distribution coefficients are listed in Table 3.

### 3.2. Bivariate Correlation Analysis Results

Sorption behavior of dinoseb is believed to significantly depend on organic carbon content, and Clay content and pH have also been reported to affect the sorption of dinoseb [32]. Correlation between the four soil properties and dinoseb adsorption capacity coefficients were assessed. Pearsons correlation coefficients (*r*) stranded for the bivariate correlation among the dinoseb *K*_*f*_ values and four soil properties (Table 4). On the basis of these data, the two-tailed parametric *t*-test was performed to investigate the significance of differences for the relations between each two variable pair [33]. Table 4 shows that correlations were all significant except that between CEC and the dinoseb *K*_*f*_ values. The dinoseb *K*_*f*_ values had highest positive correlation value with OC (*r* = 0.945), followed by Clay (*r* = 0.551) and furthermore highest negative correlation value with pH (*r* = −0.659), while there was no significant relationship between *K*_*f*_ and CEC. These results indicated that the related soil properties with the dinoseb *K*_*f*_ values were soil OC, Clay content, and pH. Moreover, it is noteworthy that correlation matrices among soil properties show several sets of relationships. The amount of OC was significantly and positively correlated with Clay content and CEC and negatively with pH at the significance level of 0.01. Similar to OC, the relationship between CEC, and Clay content was also significantly high. However, generally pH values were weakly and not significantly correlated with CEC and Clay. The results showed that not only the *K*_*f*_ is positively correlated to soil OC, CEC and Clay content, and negatively with pH, but also relationships between two soil properties are still fairly high. With the limitation of bivariate correlation, the Pearson correlation coefficients cannot demonstrate the real relationships when multicollinearity exists. 

### 3.3. Path Analysis Results

With path analysis, we can decompose the correlations into direct and indirect effects. The effects are quantified with the path coefficients (Table 5). According to the path coefficients, the sequence of direct effects to *K*_*f*_ is OC > Clay > pH > CEC. Both zero-order correlation and path analysis show OC content has a significant positive effect on *K*_*f*_, and the direct effect on *K*_*f*_ is much higher than the other three factors (path coefficient 1.056). In the zero-order correlation matrix, pH is significantly correlated with *K*_*f*_ (correlation coefficient −0.659). The path analysis shows that this correlation is mainly due to the correlation of pH with OC (path coefficient −0.662). The direct effect of pH on *K*_*f*_ is low (path coefficient −0.066). For CEC, with almost zero direct effect on *K*_*f*_, it can be considered that the moderate correlation (correlation coefficient 0.436) with *K*_*f*_ is mainly due to the contribution of collinearity between OC content and CEC. Clay content has negative direct effect on *K*_*f*_ (path coefficient −0.216), although the indirect effect due to correlation with OC is more obvious (path coefficient 0.746). Contrast to that, the correlation coefficient shows that Clay has a positive relationship with *K*_*f*_. Dinoseb is a weak acid with a pH of 4.4–4.62 [20] and is mainly in anionic form at the pH of the studied soils [34]. Therefore, it is more reasonable that its affinity to soil was negatively correlated with the content of the negatively charged clays. 

### 3.4. Stepwise Multiple-Linear Regression Results

Based on the correlation matrix in Table 4 and path analysis coefficients in Table 5, it is obvious that it is not independent between pairs of the soil properties and that makes the interpretation of multiple linear regression equations between the dinoseb *K*_*f*_ values and soil properties unreliable. The problem of multicollinearity among soil properties in linear model has been generally recognized in many studies [35, 36]. In order to overcome multicollinearity, stepwise regression, one of several standard procedures [27] for variable selection, was applied for multiple linear regression in this study. Due to the small number of correlated variables (OC, pH, CEC, Clay), the backward elimination was performed starting with all four soil properties as controlled variables and successively eliminates one at a time. And the criteria based on *t*-statistics is to remove the lowest *F*-to-remove statistic which is bigger than 0.05.

The regression coefficients and statistics summary of each prediction model of dinoseb *K*_*f*_ values depending on soil properties as developed using stepwise multiple linear regression are presented in Tables 6 and 7. 

In Table 6, the standardized coefficients (beta values) indicate the strength of the effect of the respective soil properties on dinoseb *K*_*f*_ values; that is, the larger absolute value shows the stronger effect. Zero-order correlations have been discussed in correlation analysis. Partial correlations reveal the relationship between residualized dinoseb *K*_*f*_ values and residualized soil properties, and part correlations express the correlations between residualized dinoseb *K*_*f*_ values and unaltered soil properties. 

The model 1, containing all four soil properties, explains 96.1% of the variation in dinoseb *K*_*f*_ values. However, the significant levels of CEC, pH, and Clay content indicate that some of the soil properties can be removed from the model (significant levels are 0.999, 0.497, and 0.344, resp.). According to the removal principle, the soil property with highest significant level, which is CEC, should be removed and then Model 2 is built up with the remaining soil properties; in the same way, sequential stepwise regressions eliminated pH from model 2 since pH shows the highest significant level which is bigger than 0.05 (0.000 and 0.028, resp.). In model 3, both of the remaining variables show a significant level less than 0.05, thus elimination stops (*R* ^2^ = 0.941). 

The statistics summary of each regression model is illustrated in Table 7. In addition to the three models in Table 6, Model 4 which uses only OC as a predictor is analysed. In all four models, the multiple correlations between the dinoseb *K*_*f*_ values and predictors are strong (*R* varies from 0.961 to 0.945) and decrease slightly while one specific soil property is removed from the previous model. The *R* ^2^ changes from model 1 to model 2 and from model 2 to model 3 are not significant (*P* = 0.999 and 0.481, resp.). That means that removal of CEC and pH consecutively has minor effect on the goodness of the regression, whereas removal of Clay content from model 3 results in a significant change to *R* ^2^(*P* = 0.001). That also implies the clay factor is important for dinoseb sorption in soil.

### 3.5. Model Development

Combining the results from correlation analysis, path analysis, and stepwise regression, we can conclude that the soil OC and clay contents are the most important factors affecting the dinoseb sorption in soil. Therefore, the two factors are selected as the predictors of *K*_*f*_ to build up the regression equation:

(2) $$\begin{array}{c} K_{f}=-0.175+0.067\;\text{OC}-0.10\;\text{Clay}, \end{array}$$

in which OC is the soil organic content, and Clay is the clay content. The *R* square is 0.92, that is, the variation of OC and clay in soil accounts for the 92% *K*_*f*_ variation, in which 89% variation can be explained directly by OC variation, and the other 3% can be explained by the clay content variation. The *F*-statics of the regression is 98.09, and the regression is found to be significant at *α* = 0.01.

## 4. Conclusions

A good multilinear regression was made, using all possible factors, including OC, Clay, CEC, and pH as the explanatory variables of the *K*_*f*_ values. The sequence of correlation to *K*_*f*_ was found to be OC > pH > Clay > CEC. However, The explanatory variables were not independent from each other, thus the multicollinearity may make the conclusion suspicious. With bivariate and correlation analysis and path analysis, it was found that the direct effects on *K*_*f*_ should be OC > Clay > pH > CEC. Clay, pH, and CEC are mainly correlated with *K*_*f*_ through the correlations with OC. The direct effects of pH and CEC on *K*_*f*_ are very low. The zero-order correlation matrix shows that clay was positively correlated with *K*_*f*_, but the path analysis shows that the correlation is negative. The latter is more reasonable according to the dinoseb chemical properties. The backward stepwise regression showed that pH and CEC can be removed from the prediction model. Based on these results, a more efficient regression using OC and Clay as predictors is built up. 

## Figures and Tables

**Table 1:** Main properties of dinoseb [23].

Substance	Dinoseb
Molecular formula	C_10_H_12_N_2_O_5_
Molecular weight	240.20
Water solubility (mg/L) (20 to 25°C)	50–52
Log *K*_ow_	2.29
p*K*_*a*_	4.4–4.62
*K*_oc_ (cm^3^/g)	30–130

**Table 2:** The physical and chemical properties of the soil samples.

Site	Depth cm	OC g/kg	CEC cmol/kg	pH	Clay (%)	Silt (%)	Sand (%)
	15	8.4	5.64	8.07	6.19	42.38	51.43
	30	7.63	5.57	7.99	5.01	34.62	60.37
A	55	7.26	6.87	8.04	6.93	30.46	62.61
	70	6.69	10.28	8.15	10.7	38.11	51.19
	85	14.21	10.61	7.84	4.24	34.7	61.06

	15	7.56	5.08	7.95	4.58	32.92	62.5
	30	7.67	4.98	7.93	4.29	25.41	70.3
B	55	5.33	4.78	8.36	8.21	27.61	64.18
	70	7.53	9.14	8.13	13.99	41.09	44.92
	85	12.96	10.34	8.01	4.93	18.88	76.19

	15	9.46	5.12	8.12	3.79	23.68	72.53
	30	8.94	5.16	8.01	3.49	24.22	72.29
C	55	5.97	4.82	8.21	6.3	45	48.7
	70	10.22	7.33	7.94	9.99	31.86	58.15
	85	8.83	7.32	7.89	7.32	39.23	53.45

	15	8.73	4.68	7.99	3.87	37.43	58.7
	30	7.92	4.95	7.89	4.5	37.59	57.91
D	55	6	5.03	8.1	5.33	67.85	26.82
	70	9.44	7.29	7.76	9.8	44.12	46.08
	85	11.4	7.6	7.81	10.17	40.84	48.99

OC: organic carbon content; CEC: cation exchange capacity.

**Table 3:** Averaged adsorption capacity coefficient *K*_*f*_ values of dinoseb obtained from batch experiments.

Depth (cm)	Dinoseb adsorption capacity coefficient *K*_*f*_ (cm^3^/g)				
	Site A	Site B	Site C	Site D	Mean
15	0.285	0.302	0.402	0.388	0.344
30	0.287	0.323	0.403	0.355	0.342
55	0.299	0.101	0.185	0.176	0.190
70	0.214	0.156	0.363	0.365	0.274
85	0.656	0.604	0.353	0.439	0.513

**Table 4:** Pearsons correlation coefficients (*r*) between certain soil properties and dinoseb adsorption capacity coefficients.

	*K*_*f*_	OC	pH	CEC	Clay
*K*_*f*_	1	0.945**	−0.659**	0.436	0.551*
OC		1	−0.627**	0.588**	0.706**
pH			1	−0.221	−0.321
CEC				1	0.924**
Clay					1

Asterisks denote two-tailed significance (*P < 0.05; **P < 0.01).

**Table 5:** Path analysis coefficients to *K*_*f*_ of soil factors.

	Correlation coefficient	Direct effect	Indirect effect			
			OC	pH	CEC	Clay
OC	0.945	1.056		0.041	0.000	−0.152
pH	−0.659	−0.066	−0.662		0.000	0.069
CEC	0.436	0.000	0.621	0.015		−0.200
Clay	0.551	−0.216	0.746	0.021	0.000	

**Table 6:** Regression coefficients of models when each soil factor removed^a^.

Model		Unstandardized coefficients		Standardized coefficients	*t*	Significance	Correlation		
		*β*	Standard error	Beta			Zero-order	Partial	Part
1	(Constant)	0.348	0.750		0.464	0.649			
	OC	0.064	0.008	1.056	8.285	0.000	0.945	0.906	0.595
	CEC	9.0*E* − 6	0.013	0.000	0.001	0.999	0.436	0.000	0.000
	pH	−0.063	0.090	−0.066	−0.697	0.497	−0.658	−0.177	−0.050
	Clay	−0.009	0.009	−0.216	−0.977	0.344	0.551	−0.245	−0.070

2	(Constant)	0.348	0.726		0.480	0.638			
	OC	0.064	0.007	1.056	8.645	0.000	0.945	0.908	0.601
	pH	−0.063	0.087	−0.066	−0.722	0.481	−0.658	−0.178	−0.050
	Clay	−0.009	0.004	−0.215	−2.144	0.048	0.551	−0.472	−0.149

3	(Constant)	−0.175	0.037		−4.683	0.000			
	OC	0.067	0.006	1.108	11.468	0.000	0.945	0.941	0.785
	Clay	−0.010	0.004	−0.231	−2.394	0.028	0.551	−0.502	−0.164

^a^Dependent variable: *K*_*f*_.

**Table 7:** Statistics summary of each regression model.

Model	*R*	*R* ^2^	Adjusted *R* ^2^	Standard error of the estimate	Chang statistics		
					*R* ^2^ Change	*F* Change	Significant *F* Change
1	0.961^a^	0.923	0.902	0.04287			
2	0.961^b^	0.923	0.908	0.04151	0.000	0.000	0.999
3	0.959^c^	0.920	0.911	0.04092	−0.003	0.521	0.481
4	0.945^d ^	0.893	0.887	0.04599	−0.027	150.814	0.001

^a^Predictors: (constant) Clay, pH, OC, and CEC.

^b^Predictors: (constant) Clay, pH, and OC.

^c^Predictors: (constant) Clay, OC.

^d^Predictors: (Constant) and OC.
